# Preferences, Expectations and Management Satisfaction in IBD Patients: A Cross-Sectional Questionnaire-Based Study

**DOI:** 10.3390/jcm15093266

**Published:** 2026-04-24

**Authors:** Maja Mejza, Anna Bajer, Laura Biskup, Alicja Duda, Julia Groszewska, Ewa Małecka-Wojciesko

**Affiliations:** Department of Digestive Tract Diseases, Medical University of Lodz, 90-153 Lodz, Poland; maja.mejza@stud.umed.lodz.pl (M.M.);

**Keywords:** inflammatory bowel diseases, Crohn’s disease, ulcerative colitis, patients’ expectations, reported satisfaction, questionnaire

## Abstract

**Background:** Ulcerative Colitis and Crohn’s Disease are the most common forms of inflammatory bowel disease (IBD), with increasing prevalence both globally and in Poland. Many aspects of a patients’ everyday functioning and treatment remain underexplored. **Methods:** This study recruited adult patients with IBD hospitalized in the Department of Digestive Tract Diseases of the Medical University of Lodz, Poland. The data were collected between June and July 2025 using an author-developed questionnaire assessing patients’ opinions on therapy, their expectations, and overall life satisfaction. **Results:** A total of 87 patients with IBD were included in the analysis. Overall, 59 patients (67.82%) reported strong satisfaction with their current treatment, indicating a generally positive perception of disease management. Higher treatment satisfaction was associated with patients’ perception that their preferences were respected by the gastroenterologist. Further analysis revealed significant associations between different types of treatment, disease activity, and patient-reported outcomes. Those patients who were treated with biological agents more frequently reported complete satisfaction with the treatment (41/52 vs. 18/35; *p* = 0.014), low-to-moderate disease activity (42/52 vs. 19/35; *p* = 0.016), and fewer limitations in their social lives (16/52 vs. 20/35; *p* = 0.026) compared to the remaining group. Furthermore, the study demonstrated that those patients who reported remission were less likely to have their physical activity limited (27/55 vs. 27/32; *p* = 0.002). There were significantly more patients under 50 years of age than older patients getting biological therapy (48/74 vs. 4/13; *p* = 0.045). Additionally, women in the studied group had a higher rate of IBD-related surgical interventions compared to men (15/36 vs. 9/51; *p* = 0.026). Despite the high overall satisfaction with treatment and physician communication, 20 patients (22.99%) admitted to taking additional, non-prescribed medication or dietary supplements. Similarly, 21 (24.14%) patients modified treatment regimens by discontinuing the medication intake or changing the prescribed dose. Furthermore, 57 patients (65.52%) reported that they feared the possibility of surgical intervention. Nonetheless, the majority of patients who underwent surgical treatment (22/24; 91.67%) were satisfied with the results. Reported rates of undergoing regular colorectal cancer screening were also unsatisfactory—4/37 (10.81%) patients with disease duration > 8 years, suggesting inadequate awareness. **Conclusions:** Biological treatment can positively impact multiple aspects of patients’ lives by lowering the disease’s activity. Gastroenterologists should spend more time discussing patients’ preferences and concerns, as well as explaining the colorectal cancer screening rules.

## 1. Introduction

Inflammatory bowel diseases (IBDs) are a heterogeneous group comprising Crohn’s disease (CD) and ulcerative colitis (UC). IBDs affect nearly four million people globally [1] and approximately 100,000 people in Poland [2]. The most common symptoms of CD include fatigue, abdominal pain, and loose/watery bowel movements, while patients with UC most often complain of bloody and loose/watery bowel movements and bowel movement urgency [3]. Several serious complications might occur in the course of the disease, including colonic perforation, stenosis, a toxic megacolon, fistulas, abscesses, pouchitis, and colorectal cancer (CRC) [4]. The risk of surgery for CD and UC diagnosis reaches 46.6% and 15.6%, respectively, which may be another cause of concern for the patients [5,6,7]. Currently, biologic agents are recommended for the moderate to severe cases of IBD [8,9]. Even though biologic therapy is highly effective in inducing remission [10], the risk of relapses reaches 10% at 12 months and 18% at 36 months in patients who were in remission for at least 2 years [11]. A discontinuation of treatment is associated with a 47% risk of relapse within 36 months [11]. Relapses significantly impact patients’ fitness, may lead to disability [12], depression, and psychiatric disorders [13].

Inflammatory bowel diseases have a significant negative impact on patients’ quality of life [14] as they affect many aspects of their lives, including work/education, relationships, finances, and mental health [15,16,17]. Every day, patients face multiple challenges, such as disordered sleep leading to exhaustion, the need to cope in a professional environment, a lack of public awareness or understanding, unpredictability of symptoms, and dietary restrictions. All of these may lead to the feeling of isolation, exclusion, or anxiety [18]. According to a 2021 meta-analysis, 32.1% of IBD patients experience symptoms of anxiety, and 25.2% of them experience symptoms of depression [19]. In addition, the data suggest that anxiety and depression might contribute to higher disease activity and relapse risk [20,21,22,23]. IBD also limits leisure-time activities, such as sports, which are necessary for mental and physical well-being [24]. Moreover, the disease negatively affects social functioning due to difficulties in forming and maintaining relationships [15]. Furthermore, patients are subject to stigmatization because of the character of the disease [25].

Lifestyle factors have been known to affect the IBD clinical course [26]. Recently, García-Mateo et al. [27] proved that adherence to a healthy lifestyle may lower the risk of moderate–severe IBD relapses and steroid use [28]. Matsuoka et al. [29] demonstrated that the quality of doctor–patient communication and comprehensive information provided by physicians improve patients’ compliance. Exploring patients’ preferences may improve treatment results. There is a growing need for personalized medical care, improving the patients’ satisfaction and their health-related quality of life.

This study aims to further analyze how IBD impacts patients’ daily functioning. Furthermore, it sets out to explore their preferences and attitudes regarding medications and evaluate the selected variables affecting satisfaction with the treatment.

## 2. Materials and Methods

The single-center study was conducted between June and July 2025. Patients were recruited from the Department of Digestive Tract Diseases, Medical University of Lodz, Poland. All data used in this analysis were collected from hospitalized patients. Patients over 18 years of age with an IBD diagnosis, admitted to the department during the study period, were asked to complete detailed questionnaires (Supplementary Material File S1) with the assistance of study authors, who read and clarified the questions. To be included in the study, the diagnosis of UC or CD had to be made based on clinical criteria, colonoscopy with biopsies, magnetic resonance enterography, and computed tomography, when necessary. Exclusion criteria included a disease duration shorter than one year, not being able to self-report the diagnosis, or not having a definite diagnosis. Three patients present at the hospital during the study period refused to fill in the questionnaire. In total, responses from 94 patients were obtained; however, seven patients were further excluded based on the exclusion criteria.

The questionnaire used was author-developed for the purpose of this single-center exploratory study, and its thematic scope was guided by previously published survey-based studies on IBD [30,31,32,33,34,35,36,37,38] and clinical experience. After the initial literature review-based questionnaire design, the questions and formatting were consulted with an expert gastroenterologist. However, it was not formally psychometrically validated prior to use.

**Figure 1:**
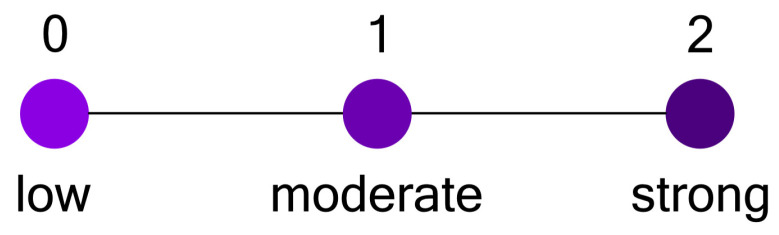
The Likert scale visualizing the scales used for reporting satisfaction or symptom severity (self-reported disease activity).

The questionnaire consisted of 64 questions. Apart from the demographic data (age, sex, place of residence, educational level, self-reported disease diagnosis, disease duration, and comorbid IBS), the questions were focused on patients’ preferences, satisfaction with treatment, and the perceived quality of communication with gastroenterologists. Moreover, the questionnaire asked about the possible discrimination and limitations in daily life (social activities, work, and physical activity) and whether the patients received support from family, support groups, or mental health professionals. Questions regarding attitude towards colonoscopies, pharmacotherapy, surgical treatment, fear of complications, and source of information about the disease were also included. Only the questions to which a straight “yes/no” or “low/moderate/high” response could be given were closed-ended. The rated responses (e.g., satisfaction) were given on a three-point Likert scale (Figure 1). The binary answers were complemented with an open field, wherever possible.

Results were extracted into Microsoft Excel and merged with the retrospective clinical data (diagnosis confirmation and the Mayo scale result on the last colonoscopy). The details that could lead to patients’ identification were omitted. The data were presented as means with standard deviations. The statistical analysis and graph creation were carried out using Python (version 3.14, Python Software Foundation, Wilmington, DE, USA) with Pandas (version 3.0.1; https://pandas.pydata.org/), NumPy (version 2.4.2; https://numpy.org/), Matplotlib (version 3.10.8; https://matplotlib.org/), Seaborn (version 0.13.2.; https://seaborn.pydata.org/), SciPy (version 1.17.1; https://scipy.org/) for univariate and statsmodels (version 0.14.6; https://www.statsmodels.org) for multivariable ordered or binary logistic regression models. The categorical variables were compared using the chi-squared test or Fisher’s exact test. Spearman’s rho was used for evaluating rank correlations. The statistical tests adopted a significant threshold of *p* < 0.05.

## 3. Results

Responses from 87 (40 with CD, 47 with UC) patients were analyzed. The disease duration was 1–30 years [mean 9 years (±7)], and the patients’ age was 19–69 [mean 38 years (±12)]. More than half of the interviewed patients were men (51 (58.62%)) and were diagnosed with UC (47 (54.02%)).

The disease activity was evaluated with Mayo scores in the last available endoscopy in 45/47 patients with UC (two patients (4.26%) had a score of 0, 11 patients (23.40%) had a score of 1, 16 patients (34.04%) had a score of 2, and another 16 patients (34.04%) had a score of 3). Time from the last endoscopy to the questionnaire ranged from 2 days after the questionnaire to 2409 days before (mean 435 days (±518)).

At the time of the questionnaire, 55 patients (63.22%) reported remission of symptoms, 26 (29.89%) perceived the disease as active, and six (6.90%) reported moderate disease activity.

Two participants finished their education at the primary school level (2.30%), 49 finished at the middle school level (56.32%), and 36 finished above middle school (41.38%). Cities were a place of current residence for 61 participants (70.11%), and 26 (29.89%) lived in the countryside. Most—61 of the patients (70.11%)—were employed, seven (8.05%) were self-employed, six (6.90%) were on disability pension, six (6.90%) were unemployed, four (4.60%) were retired, and three (3.45%) were still studying.

Among the employed patients, 33 (54.10%; 37.93% all) had less than 7 days of sick leave a year, and only six patients (9.84%; 6.90% all) had more than 30 days a year. Seventeen patients reported certified disability, and among them, five patients had a mild (5.75%) disability, and 12 had a moderate (13.79%) disability.

Alcohol consumption was reported by 32 individuals (36.78%) and tobacco use by 14 (16.09%). The rest of the patients stated complete abstinence. The baseline patients’ characteristics and preferences are presented in Table 1, Table 2, Table 3, Table 4, Table 5, Table 6 and Table 7.

Family history of IBD was reported by 17 patients (19.54%). Another three responders (3.45%) claimed that there were IBD-like symptoms in their family members, who nonetheless never got the IBD diagnosis.

Biological agents were the main treatment line in 52 patients (59.77%). The agents were as follows: infliximab—28 patients (32.18%), ustekinumab—13 patients (14.94%), vedolizumab—five patients (5.75%), and adalimumab—six patients (6.90%). Thirty-five (40.23%) patients were treated with non-biological drugs only, such as 5-ASA, steroids, and thiopurines. Additionally, 19 out of the 35 were taking upadacitinib (21.84%), three (3.45%) were taking ozanimod, and two were taking tofacitinib or filgotinib (2.30%).

There was a significant difference in the proportion of patients below and over 50 years in terms of getting biological therapy (48/74 vs. 4/13; *p* = 0.045). However, no differences were observed between disease duration groups (<5 years: 16/30, 5–10 years: 18/28, >10 years: 18/29; *p* > 0.05) or genders (25/36 vs. 27/51; *p* > 0.05). Patients treated with biologic drugs were more commonly reporting complete satisfaction with treatment than those treated with the classical medications (41/52 vs. 18/35; *p* = 0.014). They were also less likely to report high disease activity (10/52 vs. 16/35; *p* = 0.016). Moreover, on multivariable analysis, biologic medications were inversely associated with self-reported disease activity (OR = 0.298; 95% CI: 0.113–0.788; *p* = 0.015), regardless of disease duration and type, sex, age, smoking, or alcohol consumption. There were no differences between patients without higher education and university graduates in terms of getting biological therapy (35/51 vs. 17/36; *p* > 0.05).

**Figure 2:**
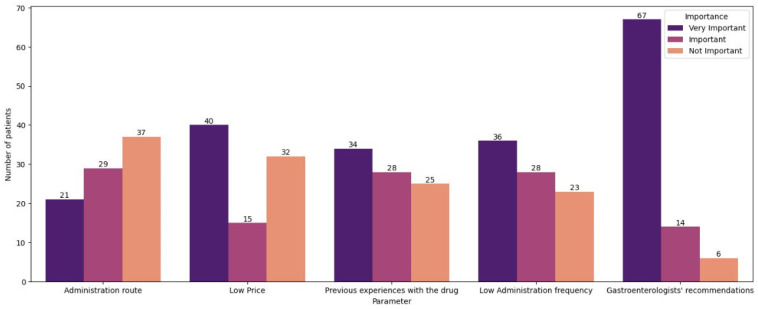
Importance of the selected parameters concerning the treatment.

Most, 59 (67.82%) patients expressed strong satisfaction (Figure 2) with their current treatment and 56 (64.37%) patients did not report any unfavorable aspects of it. The most negative aspect of the current treatment reported was the low effectiveness reported by nine (10.34%) of the patients. Moreover, the patients said that they would rather have another route of drug administration (eight patients (9.20%)) and a lower frequency of administration (eight patients (9.20%)). Only two (2.30%) patients complained of high drug prices. Other treatment limitations were low medication availability in pharmacies, a high number of medications, a non-holistic approach of gastroenterologists (i.e., not considering comorbidities, which may be affected by the drug choice), and not enough information about the drug provided by gastroenterologists. Responders were asked to rate their satisfaction with the explanations provided by their doctors from 0 to 5. Most of them (49 individuals (56.32%)) gave the highest rating of five. Lower grades were given by 16 patients (18.39%) who rated four, 11 patients (12.64%) who rated three, four patients (4.60%) who rated two, six patients (6.90%) who rated one, and one patient (1.15%) who rated 0. Participants also reported being sufficiently informed by their gastroenterologists on the mechanism of action of current medications (78 patients (89.66%)), their side effects (71 patients (81.61%)), the duration of the treatment (69 patients (79.31%)), the alternative treatment methods (60 patients (68.97%)), and disease progression (76 patients (87.36%)). Among the patients, 20 (22.99%) reported taking additional, non-prescribed medication or dietary supplements. The names of the supplements that patients could identify were sodium butyrate, triphala (Ayurvedic infusion from chebulic myrobalan), iron, magnesium, vitamin C, vitamin D3, B vitamins, essential phospholipids from soybeans (used in liver diseases), and bovine colostrum. Similarly, changing the regimen (not taking the medication or changing the dose) without consulting a gastroenterologist was also reported by 21 (24.14%) participants, mainly due to side effects or feeling worse after the regimen change (13 patients (14.94%)) or a lack of effect (five patients (5.75%)). Other causes were remission (for example, no need for 5-ASA with the biological treatment) or a lack of drug availability at the time when the drug ought to be taken.

There was a significant negative correlation between the self-reported disease activity and satisfaction with the treatment (Spearman’s rho = −0.545, *p* < 0.001). No significant differences were found between those who drank alcohol and those who did not in terms of treatment satisfaction or disease activity.

Age was inversely correlated with the feeling of being well-informed by the doctors on the medications’ mechanism of action, side effects, time to action, alternative treatment methods, and disease clinical course (Spearman’s rho = −0.276, *p* < 0.001). This effect was also noticed on multivariable analysis (OR = 0.948; 95% CI: 0.911–0.986; *p* = 0.008), regardless of the disease duration, disease type, type of treatment, age, sex, disability, or education.

Patients changing their treatment regimen without consulting a doctor were more likely to be drinking alcohol (OR = 8.425; 95% CI: 1.123–63.134; *p* = 0.038), having higher disease activity (OR = 4.200; 95% CI: 1.019–17.305; *p* = 0.047) taking additional drugs/supplements (OR = 10.519; 95% CI: 1.329–83.262; *p* = 0.026), not well-informed by their gastroenterologists (OR = 0.353; 95% CI: 0.139–0.895; *p* = 0.028), and not having medication/disease-related anxiety (OR = 0.107; 95% CI: 0.012–0.987; *p* = 0.049). This was noticeable on a multivariable logistic analysis regardless of the disease duration/type, treatment type/side effects/preferences, sex, age, education, place of residence, disability, smoking, barriers in communication with the doctor, fear of surgical intervention, or satisfaction with the current treatment.

Sixty-six patients (75.86%) stated having one or more preferred drug administration routes, with 45 preferring (51.72%) oral, 19 (21.84%) preferring intravenous, nine (10.34%) preferring subcutaneous, and four (4.60%) preferring intramuscular. The rectal route of administration was unacceptable for 29 (33.33%) participants, oral or intramuscular was unacceptable for three participants (3.45%), and intravenous was unacceptable for two participants (2.30%), while 51 (58.62%) declared that any method is acceptable for them.

For 37 individuals (42.53%), the treatment administration route is not important, whereas for 21 (24.14%), this aspect is of very high priority (Figure 1). The remaining 29 participants (33.33%) feel like this factor is of moderate importance. Conversely, drug administration frequency was very important for 36 patients (41.38%), important for 28 patients (32.18%), and not important for 23 patients (26.44%). For 40 (45.98%) patients, the price of the medication is highly important. The number of participants who defined the price as moderately important or not important was 15 (17.24%) and 32 (36.78%), respectively. Most of the interviewed patients (47; 54.02%) also stated that the time from introduction to the market was not important for them. Interestingly, patients who stated that this parameter is important (17; 19.54%) or very important (23; 26.44%) were mostly concerned with the drugs’ safety. Participants were also divided about the significance of previous experiences with the drug; however, the majority—34 patients (39.08%)—stated that it was very important to them, 28 patients (32.18%) stated that it was important, and 25 patients (28.74%) stated that it was not important. A factor considered very important or important for the highest number of participants is the gastroenterologist’s recommendation (67 patients (77.01%) and 14 patients (16.09%), respectively). This factor was not at all important to only six (6.90%) patients.

Surgical treatment of IBD was performed in 24 patients (27.59%). Out of these individuals, 22 (91.67%) were satisfied with the outcome. One patient refused to report their satisfaction. Nonetheless, fear of possible surgical treatment is experienced by 57 (65.52%) of the interviewed patients.

Women in our cohort had a higher surgery rate than men (15/36 vs. 9/51; *p* = 0.026). Additionally, there were no differences between the genders in regard to age, fear of surgery, or disease duration that could explain this phenomenon. There were, however, imbalances between disease subtype distribution: 23/36 women and only 17/51 men suffered from CD, and the remaining participants suffered from UC.

For most patients, gastroenterologists (70, 80.46%) and the internet (59, 67.82%) are the main sources of information for the disease. Family members with IBD (6, 6.90%) and books (2; 2.30%) were also important. Other mentioned sources were nurses and other patients, including patient support groups on the internet (Table 7).

Most of the participants feel that their preferences regarding treatment are respected by their gastroenterologists (75; 86.21%), who are also willing to answer their questions and provide assurance (84; 96.55%).

Only a minority of patients (19 (21.84%)) noticed barriers in communication with the gastroenterologists, such as a short consultation time (8; 9.20%), a long time lag between the scheduled appointments (3; 3.45%), and a lack of cooperation with the gastroenterologist (2; 2.30%). Individuals also complained of embarrassment when asking questions during the consultation, difficulty understanding professional language, changes in their assigned physician, disappointment with the treatment, and doctors’ impatience.

In a multivariable analysis, disease activity was inversely associated with the satisfaction of the current treatment (OR = 0.163; 95% CI: 0.074–0.360; *p* < 0.001). Moreover, a feeling that gastroenterologists respected patients’ preferences was linked to higher satisfaction (OR = 8.421; 95% CI: 1.208—58.691; *p* = 0.031). Other parameters included in the analysis—disease duration, disease type (UC/CD), age, sex, disability, type of treatment, medications’ side effects, disability, feeling well-informed about the disease by the physician, or barriers in communication with the gastroenterologists—were not found to be significantly connected to the satisfaction in this cohort.

Only nine patients (10.34%) reported experiencing disease-related discrimination in everyday life, particularly regarding the lack of toilet accessibility in public spaces (6; 6.90%), social exclusion at work (3; 3.45%), and social exclusion in everyday life (2; 2.30%). When asked directly, 36 (41.38%) patients reported that the disease limits their social contacts. The most common issues are the obstacles associated with leaving the house (34, 39.06%), going on trips (3; 3.45%), dietary restrictions (4; 4.60%), disease-related fatigue (4; 4.60%), or alcohol avoidance (1.15%). Furthermore, 54 (62.07%) patients reported that the disease limits their physical activity, and 67 (77.01%) patients reported that stress is impacting the disease course. Less than a fifth of patients (15 (17.24%)) reported regularly consulting psychologists or psychiatrists. Nearly all of them (14 (93.33%)) believed in the benefit of this care. Moreover, 12/72 (16.67%) patients, not yet consulted by those specialists, found that it could be helpful for them. Most patients (81; 93.10%) reported getting support from their family, but only three (3.45%) were involved in formal patient support groups.

Patients treated with the biological therapy are less likely to limit social contacts than patients using other drugs (16/52 vs. 20/35; *p* = 0.026). However, the type of therapy does not impact the physical activity avoidance (28/52 vs. 26/35; *p* = 0.089). People who reported remission of symptoms were less likely to limit their physical activity than those currently complaining of disease symptoms (27/55 vs. 27/32; *p* = 0.002).

As many as 33/37 (89.19%) patients with a disease duration of over 8 years denied undergoing CRC screening (Table 7). However, when directly asked about a colonoscopy, the majority confirmed undergoing it regularly (28/37, 75.68%). Only 2/37 (5.41%) patients reported refusing a colonoscopy, mainly when anesthesia was not provided. There is a possibility that the screening was performed, but we have not directly discussed it with the patient.

## 4. Discussion

The mean age of patients in our study was 38 years, which is consistent with national data, implying that the highest prevalence of IBD in Poland is within the 30–44-year age group [2]. The mean age is, however, lower than reported in the global IBD population [39], which may have affected the results. The study cohort included a slightly higher number of men (58.62%) than women. This male predominance corresponds with both Polish and international data, which indicate a higher prevalence of IBD among men [2,39].

Most patients (59.77%) in our cohort were treated with biologic therapy. However, our questionnaire was targeted at hospitalized patients. This resulted in an important selection bias. More patients treated with biologic drugs reported complete satisfaction with treatment than those treated with the classical medications. The use of biologics was related to better quality of life parameters, including lower self-reported disease activity or not having to limit social contacts. Similar positive outcomes can be found in the literature [40,41,42,43,44]. Steenholdt et al. demonstrated that patient quality of life, assessed by the Short Inflammatory Bowel Disease Questionnaire (SIBDQ), improves rapidly following the initiation of therapy with vedolizumab [40]. Significant improvements compared to the baseline were observed both after one year of treatment and at the time of treatment failure (defined as drug discontinuation, necessity for surgery, or requirement for rescue therapy) [40]. This indicates that even when vedolizumab did not achieve long-term maintenance, patients still experienced a clinical gain compared to their highly active disease state at baseline. However, the authors also reported that fatigue did not improve as dynamically as other quality of life parameters. This could be one of the reasons why a connection between this treatment and physical activity was not observed.

Across the literature, biologic therapies are reported to significantly reduce disease activity in patients with IBD. Biologic agents were shown to be effective in inducing clinical remission and reducing inflammation [45]. Moreover, meta-analyses demonstrated that biologics significantly increase rates of mucosal healing—an important marker of disease activity, which has been associated with better long-term outcomes in both CD and UC [46]. Real-world effectiveness data also indicate that biologic therapies lead to clinically significant improvements in both symptom control and objective markers of disease activity [10].

In our study, significantly more patients younger than 50 years received biologic therapy compared with those aged 50 years and older, with no significant difference observed between men and women. This age-related disparity may be partially attributable to the predominance of younger patients in our cohort. Mahlich et al. also demonstrated that in a Japanese population, younger age (≤40 years) was associated with better odds of biologic treatment compared to older age (>65 years) (OR = 0.24; 95% CI: 0.09–0.64) [47]. In the study, patients aged ≤40 years accounted for 44.89% (79/176) of those receiving biologics, while individuals older than 65 years represented only 4.55% (8/176). Similarly, Kucha et al. [48] reported the highest rates of biologic therapy among Polish patients aged 10–19 years, with biologics prescribed to 21.7% of patients with CD and 6.5% of those with UC. Patients over 70 years were the least likely to receive such treatment, with usage rates below 1% for both diseases [48]. This pattern aligns with observations from previous studies and may reflect multiple factors influencing treatment decisions in older individuals, including healthcare system factors. In the Polish healthcare system, access to biologic therapy for IBDs is regulated through dedicated National Health Fund drug programs for Crohn’s disease (B.32) and ulcerative colitis (B.55) and depends on meeting specific eligibility criteria regarding diagnosis and unsatisfactory responses to conventional treatment [49,50,51]. Elderly patients often have a higher burden of comorbidities and are more susceptible to infections, which increases risks associated with immunosuppressive biologic therapies [52,53]. Furthermore, the elderly are frequently underrepresented in clinical trials, limiting evidence on the efficacy and long-term safety of biologics in this age group [54]. Age-related changes in immune function, polypharmacy, and the tendency of later-onset IBDs to present with a milder disease phenotype may also contribute to favoring conventional therapies over biologics [55].

Regarding gender distribution, our study observed no significant gender-based differences in the prevalence of biologic treatment in the men and women who were recruited. This stands in contrast to recent data from the Polish National Health Fund, which revealed significant gender disparities: females were less likely than men to receive biological treatment in both Crohn’s disease (6.7% [790/11,713] vs. 9% [1073/11,861]) and ulcerative colitis (1.4% [509/35,964] vs. 1.8% [665/37,271]) [48]. Similarly, we did not observe a correlation between disease duration and the use of biologic therapy in our cohort. However, Mahlich et al. reported that disease duration was a key determinant in their study, where patients’ disease duration of >15 years had a substantially higher likelihood of biologic use compared with those with a disease duration of <2 years (OR = 4.16; 95% CI: 1.80–9.56) [47].

In our study, patients frequently reported dissatisfaction with the route of administration or administration frequency, which may be attributed to the hospital-based administration of medications. As a result, patients often need to take formal sick leave, which may increase their professional and financial burden. Furthermore, long travel distances could also be a significant concern, especially for those living in rural areas. This geographic barrier is a direct result of the centralization of the Polish biological treatment programs. Access is limited to specialized centers that meet strict criteria, including the presence of certified gastroenterologists and extensive institutional experience in IBD management. Consequently, the need to commute to those selected hospitals remains a major challenge for patients living far from the academic centers. Addressing the access to multidisciplinary teams and specialist consultations was already proposed as a part of the larger-scale reforms needed for improving the quality of care for IBD patients in Poland [44].

While the high cost of biologics can limit their use globally [56,57,58], in Poland, biological therapy is fully refunded by the National Health Fund. This likely explains why socioeconomic factors, such as higher education or employment, did not increase the likelihood of receiving biological treatment in our study. Interestingly, we found that patients without a university degree received biologics more frequently. This suggests that in Poland, access to advanced therapy is based on medical necessity rather than social or economic status.

In our cohort, most of the patients (75.86%) reported having a preferred route of administration. More than half of the patients (51.72%) indicated a preference for oral drug administration, which is consistent with previous studies [59,60,61]. This highlights the need for developing new oral-targeted drugs. Although orally administered small molecules, such as Janus kinase inhibitors, are already used in clinical practice [62,63], the oral delivery of large-molecule biologic drugs remains challenging because of the enzymatic degradation within the gastrointestinal tract. In this context, oral biologic nanomedicines represent a promising therapeutic innovation [64,65,66,67]. In parallel with advances in drug delivery, emerging molecular targets involved in intestinal inflammatory signaling are also being explored; recent preclinical data identified N4BP3 as a potential therapeutic target through the activation of the TLR4/NF-κB axis [68]. Unlike traditional formulations, nanocarriers can protect protein-based agents from gastric degradation and facilitate a targeted delivery to inflamed intestinal tissue [69]. Such site-specific delivery has the potential to increase local drug concentration while minimizing systemic exposure and potential side effects [65,66,67].

Our study found that while the drug administration route was important, even more patients were concerned with having a low administration frequency. This observation can be supported by multiple studies reporting a preference for less frequent regimens [61,70,71]. For instance, a Taxonera et al. study on patients switching adalimumab regimens from 40 mg weekly to 80 mg every other week (EOW) found that 74% of patients preferred the less frequent EOW schedule [61]. Similarly, Fiorino et al. have confirmed this trend, with more than 60% of patients favoring an injection every other week over a weekly administration [70].

In our survey, nearly one quarter of patients reported using additional over-the-counter products (drugs or dietary supplements) that were not prescribed by gastroenterologists. This is consistent with the published data indicating that self-initiated adjunctive therapies and dietary changes are common among patients with IBD, with studies estimating that up to half of patients use some form of complementary and alternative medicine (CAM) at some point during the disease course [72,73,74]. In a longitudinal, population-based inception cohort, 30% of patients reported CAM use at some point since diagnosis, and 7.5% reported current use, supporting that adjunctive non-prescribed therapies represent relevant real-world behavior in IBDs [75].

In our study, only a minority of patients (27.59%) underwent surgical intervention for the disease. It is important to point out that surgical history related to the disease was more prevalent in women than in men. A large meta-analysis from 2024 suggested the opposite—that males generally require more IBD surgeries (RR: 1.10, 95% CI: 1.01–1.20) [76]. Notably, we observed that CD was more prevalent in women than in men in our cohort. This could explain our observation, as patients with CD often require more disease-related surgeries [77,78].

In our survey, most patients reported fear of surgical intervention (65.52%), even though those who underwent it were satisfied with its effectiveness in symptom management (91.67%). This pattern is consistent with published data indicating that IBD patients often perceive surgery as a “last resort” and report substantial preoperative concerns [6,76,79]. In the study by Spinelli et al., 80% of respondents considered surgery the last option, and 73% cited fear of surgical complications; nevertheless, most patients reported coping with their stoma better than expected or as expected [6]. Importantly, the prospect of an ileostomy has been described as one of the most significant concerns among IBD patients [76,79].

Alcohol consumption is connected to worsening IBD symptoms [80]. Regardless, 36.78% of our cohort reported consuming it at least occasionally. Additionally, alcohol can change the effects of many medications, leading to decreased effectiveness and satisfaction from treatment. In other IBD cohorts, alcohol consumption is also commonly reported. In the Swiss Inflammatory Bowel Disease Cohort Study, 41.3% of patients reported regular alcohol use (≥1 drink per week) [81]. Similarly, a U.S. survey in patients with inactive IBD reported that 62% were current drinkers; this proportion was broadly comparable to estimates reported for the general U.S. population [81].

Smoking is considered an important risk factor for CD relapses [82], and the reverse relation can be noticed in the case of UC [82]. In our cohort, only 16.09% of patients admitted to smoking, which is less than in the general Polish population [82], suggesting patients may stop smoking after the IBD diagnosis. In other IBD cohorts, the prevalence of current smoking varies but is frequently reported at levels comparable to or higher than in our sample. In a UK outpatient survey (n = 465), 15.7% of patients were current smokers, with smoking being more common in CD than in UC (21.5% vs. 7.7%) [83]. In the Swiss IBD Cohort Study (n = 1770), 29% of patients were current smokers, with substantial differences by IBD subtype and sex [84]. In a population-based inception cohort from Hungary, 47.2% of patients with CD were smokers at the time of diagnosis [85]. Published inception cohort studies show that smoking prevalence in IBD differs substantially across countries and has changed over time [86].

In our cohort, 19.54% of respondents reported IBD occurrence among family members; this should be interpreted in the context of self-reported survey data, whereas clinically assessed cohorts typically report 8–12% [87]. Nevertheless, higher proportions have been described in selected populations, particularly in pediatric IBD patients, where Ruban et al. reported a positive family history in 25.2% of 325 children with IBDs [87].

Only 21.84% of patients complained of significant barriers in communication with gastroenterologists. The biggest obstacle was a limited time during the consultation (9.20%). Similar results were pointed out by Huisman et al. [88], who also attributed inadequate information given during consultations to time limits. Importantly, older patients were less frequently feeling well-informed about their disease. This should be explored deeper in further studies of larger and more diverse populations, as in this cohort, patients over 50 years of age were a minority.

Overall, only a small proportion of patients reported having experienced discrimination due to their disease (10.34%), a rate substantially lower than that observed in Korean [15] or American populations [89,90,91], where estimates range from 27% to 47%. This difference may be attributable to the Polish legal and social context, in which disease-related sick leave (L4) is explicitly regulated by labor law for individuals employed under standard employment contracts. Taking medical leave is a common, socially accepted, and legally sanctioned practice, which is likely to reduce workplace stigma and, consequently, patients’ perceptions of discrimination. Indeed, a systematic review conducted by Keefer and Taft has shown that being denied sick leave for outpatient care and appointments was a major source of workplace discrimination in patients with IBDs [92].

The single factor most commonly reported as negatively impacting patients’ quality of life was access to public toilets (6.90%). Similar concerns have been reported by multiple authors, who have demonstrated that inadequate restroom accessibility significantly affects quality of life and overall well-being across both adult and pediatric populations [93,94,95].

Previous studies have consistently shown that psychosocial factors significantly influence life satisfaction among patients with IBDs. Depression has been shown to correlate negatively with life satisfaction, while social support shows a strong positive association with it [96]. More recent evidence further indicates that social support remains a key determinant of life satisfaction, with its effects being moderated by psychological resilience and depressive symptoms [97]. In our cohort, most patients reported getting help from family (93.10%), but participation in patient support groups was infrequently reported (3.45%). However, we did not evaluate other forms of interpatient support. For instance, previous studies reported the use of online forums for learning about IBDs and finding empathy from other people with similar disease-related problems [98,99,100]. We have not assessed the adequacy or quality of patients’ social networks. Therefore, direct comparisons with studies focusing on broader psychosocial constructs remain limited.

Physical activity has also been identified as an important factor influencing disease-specific and patient-reported outcomes in IBDs, including depression, pain, fatigue, sleep disturbance, and satisfaction with social roles and activities [101]. Consistent with these findings, patients in our study who reported disease remission were significantly less likely to restrict their physical activity, suggesting an association between better disease control and higher functional capacity.

Interestingly, only a small proportion of patients with IBDs in our cohort reported having consulted a psychiatrist or psychologist. Despite this limited uptake, almost all patients who underwent such consultations reported satisfaction with this decision and perceived psychological support as beneficial for both their overall well-being and disease management. This fact seems especially important, considering that most of our cohort considered stress as a factor negatively impacting their symptoms. The British Society of Gastroenterology has previously recommended psychological therapies as a potential adjunct in the management of IBD symptoms [102], while emphasizing that this recommendation is based on very low-quality evidence.

According to the 2023 Polish guidelines for ulcerative colitis, the first surveillance colonoscopy should be performed 8 years after diagnosis [103]. However, as many as 89.19% of patients with a disease duration exceeding 8 years denied undergoing CRC screening. However, when directly asked about a colonoscopy, the majority confirmed undergoing it regularly. This discrepancy may indicate limited awareness of what constitutes CRC surveillance in IBDs and highlights the potential confusion between colonoscopies that are performed for disease assessment and colonoscopies that are performed specifically for oncological surveillance. In Crohn’s disease, an ileocolonoscopy, with terminal ileum assessment and biopsies, is recommended as the primary endoscopic investigation and remains an important tool to evaluate disease activity; therefore, patients may undergo repeated endoscopy without recognizing it as cancer-focused surveillance [104]. Our findings can be supported by the work of Khan et al. [28], who showed that despite the general (88%) knowledge of increased CRC risk, only 20.7% of British patients saw colonoscopy as the best screening tool. The majority (88%) of patients were also unaware of the optimal screening initiation time. Nevertheless, the information on the need for colorectal cancer screening for IBDs was not prevalent in our patients, which needs to be improved.

Most of the 37 patients with long disease duration did not report issues with undergoing a colonoscopy. Even fewer reported ever refusing it (5.41%). This is generally compatible with data by Braithwaite et al. [104], which showed that even though patients generally view colonoscopy as uncomfortable, they would still accept it according to the current surveillance guidelines. At the same time, reluctance to undergo a surveillance colonoscopy—particularly during clinical remission—may persist in a subset of patients due to concerns about procedure-related symptom worsening; the available evidence suggests that some individuals may experience transient symptom exacerbation shortly after bowel preparation for a colonoscopy, although this does not necessarily translate into a sustained disease relapse in most cases [105,106].

Study limitations should be acknowledged. The questionnaire was author-developed and was not formally psychometrically validated; therefore, the findings should be interpreted as exploratory. A further limitation is that the study included only 87 Polish patients treated at a single tertiary hospital, and all data were collected from hospitalized patients, which may have introduced a selection bias and may limit the generalizability of the findings to other settings, populations, and regions. This study was not designed to detect statistically significant differences between treatment types or screening strategies, but rather to highlight the wide range of needs and challenges experienced by patients with IBD that may inform future studies. The methodological novelty of this study lies in the use of a single exploratory questionnaire integrating multiple patient-centered domains that are often assessed separately in the literature, including daily life, treatment satisfaction, preferences regarding route and frequency of administration, self-directed treatment changes, use of non-prescribed products, attitudes toward surgery, and colorectal cancer screening awareness.

Future research should focus on conducting multicenter studies with larger and more diverse populations to improve the generalizability of findings beyond a single-center setting. Additionally, it would be valuable to develop and validate a standardized questionnaire to ensure its reliability and consistency for broader clinical use. Longitudinal approaches could also be implemented to track changes in patient preferences and satisfaction over time, particularly during the transition between therapeutic modalities or after surgical interventions. Furthermore, specific attention should be directed toward investigating the barriers preventing older patients from accessing biological therapies to ensure more equitable treatment across all age groups. There is also a clear need to evaluate the efficacy of structured educational programs concerning colorectal cancer screening and pre-operative counseling in Poland to effectively reduce patient anxiety and stigma. Finally, future studies should explore the clinical benefits of integrating routine psychological support into comprehensive inflammatory bowel disease management to further improve patient-reported outcomes.

## 5. Conclusions

Biological treatment has the potential to improve patients’ disease activity control. We demonstrated that patients on biologics report milder symptoms and are less prone to limiting social interactions. In our cohort, younger patients were the majority of those taking biologics. Research into the barriers that prevent older patients from accessing biological therapy is advised. Patients who underwent surgery for IBD were generally satisfied with the results. Nonetheless, most patients still fear surgical interventions and the stigma surrounding them. This confirms that complex interventions and a holistic approach to IBD patients are crucial for treatment optimization. The pros and cons of the surgery should be explained in detail to the patients, even before there is any indication of anxiety reduction. In our cohort, women had a higher rate of IBD-related surgical interventions. Discussing possible options is especially important considering how diverse patients’ opinions are on the preferred route of treatment and what the most important factor is for treatment to meet their needs. Generally, the treatment with oral drugs, which are less often administered in the hospital, should be considered for optimal patient satisfaction. The time for discussion of the disease with the patients should be extended. Consulting psychiatrists or psychologists may be useful for patients in need of such interventions. It is, however, not clear whether such interventions should be routinely advised. Undoubtedly, a comprehensive approach to treatment by gastroenterologists is needed, as patient satisfaction is correlated with gastroenterologists integrating their preferences into treatment protocols. The patient support groups should be mentioned in the discussion with the patients to bring more awareness to their existence. Moreover, clinicians need to pay more attention to informing patients about the CRC screening rules in their condition.

## Figures and Tables

**Table 1:** The baseline characteristics of patients with IBD.

Characteristics	Patients (n = 87)
Sex, n (%)	
Male	51 (58.62%)
Female	36 (41.38%)
Age, years, n (%)	
18–24	13 (14.94%)
25–49	61 (70.11%)
50–69	13 (14.94%)
Disease, n (%)	
CD	40 (45.98%)
UC	47 (54.02%)
Mayo 0	2 (2.30%)
Mayo 1	11 (12.64%)
Mayo 2	16 (18.39%)
Mayo 3	16 (18.39%)
Colonoscopy not available	2 (2.13%)
Comorbid IBS, n (%)	4 (4.60%)
Disease duration, n (%)	
1–4 years	30 (34.48%)
5–10 years	28 (32.18%)
>10 years	29 (33.33%)
Self-reported disease activity level, n (%)	
Not active	55 (63.22%)
Moderate	6 (6.90%)
Active	26 (29.89%)
Educational level, n (%)	
Primary school	2 (2.30%)
Middle school	49 (56.32%)
College and above	36 (41.38%)
Current residence, n (%)	
City	61 (70.11%)
Countryside	26 (29.89%)
Employment, n (%)	
Employed	61 (70.11%)
Self-employed	7 (8.05%)
Unemployed	6 (6.90%)
Disable pension *	6 (6.90%)
Retired	4 (4.60%)
Student	3 (3.45%)
Certified disability, n (%)	
Mild	5 (5.75%)
Moderate	12 (13.79%)
Severe	0 (0.00%)
No disability	70 (80.46%)
Substance use, n (%)	
Alcohol	32 (36.78%)
Tobacco (smoking)	14 (16.09%)
Family history of IBD, n (%)	
Yes	17 (19.54%)
No	67 (77.01%)
Not confirmed	3 (3.45%)

* a person unable to work, who receives disability pension. CD—Crohn’s disease, IBS—irritable bowel syndrome, and UC—ulcerative colitis.

**Table 2:** Patients’ attitudes towards the current treatment.

Characteristics	Patients (n = 87)
General satisfaction with the current treatment, n (%)	
Strong	59 (67.82%)
Moderate	22 (25.29%)
Low	6 (6.90%)
Unsatisfactory aspects of the current treatment, n (%)	
None	56 (64.37%)
Low effectiveness	9 (10.34%)
High administration frequency	8 (9.20%)
Inconvenient administration route	8 (9.20%)
High drug price	2 (2.30%)
Other	4 (4.59%)
Enough information from gastroenterologist, depending on information type, n (%)	
Mechanism of action of the current treatment	78 (89.66%)
Side effects of the current treatment	71 (81.61%)
Time to action of the current treatment	69 (79.31%)
Alternative treatment methods	60 (68.97%)
Disease clinical course	76 (87.36%)
Taking additional medication or supplements, not recommended by a gastroenterologist, n (%)	
Yes	20 (22.99%)
No	67 (77.01%)
Changing the regimen without consulting a gastroenterologist, n (%)	
Yes	21 (24.14%)
Reason for regimen change	
Side effects or worsening	13 (14.94%)
Lack of effects	5 (5.75%)
Other	3 (3.45%)
No	66 (75.86%)

**Table 3:** Preferences for the route of administration.

Patients’ Preferences on IBD Drugs	Patients (n = 87)
Preferred treatment administration route, n (%)	
Yes	66 (75.86%)
P.o	45 (51.72%)
I.v.	19 (21.84%)
S.c.	9 (10.34%)
I.m.	4 (4.60%)
No	21 (24.14%)
Unacceptable treatment administration route, n (%)	
Yes	36 (41.38%)
Rectal	29 (33.33%)
P.o.	3 (3.45%)
I.m.	3 (3.45%)
I.v.	2 (2.30%)
No	51 (58.62%)

**Table 4:** The surgical history of patients with IBD.

Characteristics	Patients (n = 87)
Surgical history related to IBD, n (%)	
Yes	24 (27.59%)
Satisfaction with the surgery, n (%)	
Strong	14 (16.09%)
Moderate	8 (9.20%)
No satisfaction or no answer	2 (2.30%)
No	63 (72.41%)

**Table 5:** Information on the disease and patient–gastroenterologist relations.

Characteristics	Patients (n = 87)
Main sources of information about the disease, n (%)	
Gastroenterologists	70 (80.46%)
Internet	59 (67.82%)
Family members diagnosed with IBD	6 (6.90%)
Other patients	6 (6.90%)
Books/Brochures	4 (4.60%)
Nurses	1 (1.15%)
Gastroenterologists respect patients’ preferences in treatment, n (%)	
Yes	75 (86.21%)
No	12 (13.79%)
Gastroenterologists are open to discussing patients’ doubts, n (%)	
Yes	84 (96.55%)
No	3 (3.45%)
Barriers in communication with gastroenterologists	
Yes	19 (21.84%)
Lack of time during consultation	8 (9.20%)
Long time lag between the appointments	3 (3.45%)
Lack of understanding from the doctors	2 (2.30%)
Other	6 (6.90%)
No	68 (78.16%)

**Table 6:** The impact of IBD on patients’ quality of life and sense of security.

Characteristic	Patients (n = 87)
Getting help from family, n (%)	
Yes	81 (93.10%)
No	6 (6.90%)
Patients’ support groups involvement, n (%)	
Yes	3 (3.45%)
No	84 (96.55%)
Getting help from mental health professionals, n (%)	
Yes	15 (17.24%)
No	72 (82.76%)
A feeling of discrimination, n (%)	
Yes	9 (10.34%)
Toilet accessibility	6 (6.90%)
Work loss/instability	3 (3.45%)
Social and romantic relationships	2 (2.30%)
No	78 (89.66%)
Limiting social contacts, n (%)	
Yes	36 (41.38%)
Reasons	
Problems with going out	34 (39.08%)
Dietary restrictions	4 (4.60%)
Fatigue	4 (4.60%)
Problems with going on trips	3 (3.45%)
Alcohol	1 (1.15%)
No	51 (58.62%)
Limiting physical activity, n (%)	
Yes	54 (62.07%)
No	33 (37.93%)

**Table 7:** Acceptance of cancer screening in patients with disease duration over 8 years.

Characteristics	Patients with Disease Duration over 8 Years (n = 37)
Reported undergoing CRC screening, n (%)	
Yes	4 (10.81%)
No	33 (89.19%)
Issues with undergoing a colonoscopy, n (%)	
Yes	8 (21.62%)
No	28 (75.68%)
N/A *	1 (2.70%)
History of colonoscopy refusals, n (%)	
Yes	2 (5.41%)
No	34 (91.89%)
N/A *	1 (2.70%)

* not undergoing a colonoscopy after colectomy.

## Data Availability

Data generated and analyzed for the study are not publicly available.

## Supplementary Materials

The following supporting information can be downloaded at: https://www.mdpi.com/article/10.3390/jcm15093266/s1, Supplementary Material File S1.

## Abbreviations

The following abbreviations are used in this manuscript:

IBD	Inflammatory Bowel Disease
CD	Crohn’s Disease
UC	Ulcerative Colitis
IBS	Irritable Bowel Syndrome
CRC	Colorectal Cancer
CAM	Complementary and Alternative Medicine
EOW	Every Other Week
