# Supplementary material for: Preferences, Expectations and Management Satisfaction in IBD Patients: A Cross-Sectional Questionnaire-Based Study
Source: J Clin Med. 2026 Apr 24;15(9):3266. doi: 10.3390/jcm15093266 (PMC13163585; doi:10.3390/jcm15093266)
Supplement: Supplementary file 1 [file jcm-15-03266-s001.zip › jcm-4217934-supplementary.pdf]

**Age:**<br>_____<br><br>**Educational level:**<br>Primary school / Middle school / College or above	**Sex:**<br>M / F<br><br>**Current residence:**<br>Countryside / City
**Disease:**<br>UC – Ulcerative colitis /<br>CD – Crohn's disease<br><br>**Disease duration:** ____________<br><br>**Comorbid IBS:** YES / NO	**Employment:**<br>Employed / Self-employed / Unemployed / Disable pension / Retired / Student<br><br>**Formal sick leave (days):**<br>____
**Experience of discrimination:** YES / NO<br><br>**If yes, in what circumstances?**<br>__________________________________	**Limiting social interactions: YES / NO**<br><br>**If yes, in what circumstances?**<br>__________________________________
**Getting support from family:** YES / NO<br><br>**If yes, in what circumstances do You need the support?** ________________________________<br><br>**Family history of IBD:** YES / NO	**Participation in patients' support groups:** YES / NO<br><br>**If yes, what benefits do you derive from these groups?**<br>______________________________________<br><br>**If not, why?** ___________________________
**Certified disability:** YES / NO<br><br>**If yes, then specify the level:**<br>Severe / Moderate / Mild	**Consulting a psychologist/psychiatrist?** YES /NO<br><br>**Do you find such consultations are/could be useful?**<br>YES / NO
**Smoking:** YES / NO	**Drinking alcohol:** YES / NO
**Do you believe stress can impact the disease course?** YES / NO	**Limiting physical activity:** YES / NO
**Satisfaction with current treatment:**<br>Strong / Moderate / Low	**What time do you consider acceptable for improvement after starting treatment?**<br>____
**Does the treatment you are taking cause any side effects?** YES / NO<br><br>**If so, what side effects have you noticed?**<br>____	**Do you feel your doctor has adequately informed you about:**<br>- The mechanism of action of the medications you are taking: YES / NO<br><br>- Side effects of the medications you are taking: YES / NO<br><br>- Duration of treatment: YES / NO<br><br>- Alternative treatment options: YES / NO<br><br>- Disease progression: YES / NO
**Are you concerned about complications of the disease or treatment?** YES / NO<br><br>**If so, what are you concerned with?**<br>____	
**Are you currently taking any additional treatments not recommended by your doctor?**<br>YES/NO<br><br>**If so, what are they?** ________________________	**What in your life is the best indicator of treatment effectiveness?**<br>Improved mood / Opportunities for socializing / professional development / travel / Other<br>(____)

**Which of the following routes of medication administration do you prefer?**<br>Oral / Rectal / Subcutaneous / Intramuscular / Intravenous<br><br>**If you would like to change your current treatment, what would it be?**<br>Medication frequency / Route of administration / Price / Availability / Effectiveness in reducing symptoms /<br>Other (______________________)<br><br>**Why?**<br>________________________________	**How important are the following to you:**<br><br>**- Frequency of medication:**<br>Very important / Moderately important / Not important<br>**- Route of administration:**<br>Very important / Moderately important / Not important<br>**- Price of the medication:**<br>Very important / Moderately important / Not important<br>**- Period of drug availability on the market:**<br>Very important / Moderately important / Not important<br>**- Previous experience with the medication:**<br>Very important / Moderately important / Not important<br>**- Doctor's recommendation of the medication:**<br>Very important / Moderately important / Not important
**Have you ever discontinued/modified treatment on your own?** YES / NO<br><br>**If so, what was the reason?**<br>Lack of financial resources / Side effects / No improvement / Other (_________________)	**What symptoms most impact your quality of life?**<br>Urgent need to use the bathroom / Diarrhea / Abdominal pain / Fatigue / Weight loss / Nausea / Bloating / Other (____________________________________)
**What is your main source of information about your illness?** Doctor / Family / Support groups / Internet / Other (please specify)<br><br>**Do you feel that your doctor takes your treatment preferences into account?** YES / NO<br><br>**Do you feel that you have the opportunity to ask your doctor questions?** YES / NO<br><br>**What is your main barrier to communicating with your doctor?** Specialized language / Lack of time for discussion / Fear of being judged / Other (_____________________________)	**Have you been offered oncological surveillance due to your disease?** YES / NO<br><br>**Does regular colonoscopy as part of surveillance pose a problem for you?** YES / NO<br><br>**Have you ever refused a follow-up colonoscopy?** YES / NO<br><br>**If so, why?** ________________________________<br><br>**Has surgery been performed for your disease?** YES / NO<br><br>**If so, how would you rate its effectiveness?** Very good / Good / Poor<br><br>**Does the possibility of such treatment make you anxious?** YES / NO
